# Transgenerational, Dynamic Methylation of Stomata Genes in Response to Low Relative Humidity

**DOI:** 10.3390/ijms14046674

**Published:** 2013-03-26

**Authors:** Penny J. Tricker, Carlos M. Rodríguez López, George Gibbings, Paul Hadley, Mike J. Wilkinson

**Affiliations:** 1 School of Biological Sciences, Philip Lyle Building, University of Reading, Whiteknights, Reading RG6 6BX, UK; E-Mails: j.g.gibbings@reading.ac.uk (G.G.); p.hadley@reading.ac.uk (P.H.); 2 Institute of Biological, Environmental and Rural Sciences, Edward Llywd Building, University of Aberystwyth, Aberystwyth, Ceredigion SY23 3DA, UK

**Keywords:** environmental epigenetics, transgenerational methylation, water stress, siRNAs, stomata, *SPEECHLESS*, *FAMA*

## Abstract

Transgenerational inheritance of abiotic stress-induced epigenetic modifications in plants has potential adaptive significance and might condition the offspring to improve the response to the same stress, but this is at least partly dependent on the potency, penetrance and persistence of the transmitted epigenetic marks. We examined transgenerational inheritance of low Relative Humidity-induced DNA methylation for two gene loci in the stomatal developmental pathway in *Arabidopsis thaliana* and the abundance of associated short-interfering RNAs (siRNAs). Heritability of low humidity-induced methylation was more predictable and penetrative at one locus (*SPEECHLESS, entropy* ≤ 0.02; χ^2^ < 0.001) than the other (*FAMA, entropy* ≤ 0.17; χ^2^ ns). Methylation at *SPEECHLESS* correlated positively with the continued presence of local siRNAs (*r*^2^ = 0.87; *p* = 0.013) which, however, could be disrupted globally in the progeny under repeated stress. Transgenerational methylation and a parental low humidity-induced stomatal phenotype were heritable, but this was reversed in the progeny under repeated treatment in a previously unsuspected manner.

## 1. Introduction

The sessile existence of plants and their often limited seed dispersal means that most individuals are likely to experience broadly similar growth conditions to their (maternal) parents. However, stressful growth environments arise periodically in many locations and may persist for one, a few, or many generations. Anthropomorphically-driven global climate change is likely to increase the frequency and extent of such year-to-year climatic variance in the coming decades [1,2]. Viewed in this context, it is important to gain a better understanding of the key physiological features that enhance the resilience of plants to withstand trans-seasonal fluctuations in the growing environment. The concept of the “soft inheritance” [3] of gene expression patterns through epigenetic mechanisms provides an attractive framework for explaining how alterations in progeny phenotypes could be driven by a memory of the environmental exposure of the parent. Physiological evidence of “transgenerational hardening” [4] has been documented in both annual and perennial plants [5–7] but the molecular epigenetic mechanisms underpinning these effects have yet to be resolved [8]. There are, nevertheless, several candidate epigenetic regulatory systems that may play some part in mediating these transgenerational responses. These include transgenerational, stress-induced somatic homologous recombination [9], altered gene expression patterns [3] and altered DNA methylation patterns [10]. In *Arabidopsis*, however, it has been suggested that there is an epigenomic re-setting event during gametogenesis that cleanses the genome of epigenetic DNA and histone modifications [11]. During this process, it is hypothesised that some epigenetic modifications are stably re-introduced, essentially back to the embryonic start point [12], and that other, stress-induced modifications are inherited stochastically, possibly through inefficiencies of the resetting process, and generally have little endogenous effect on the genome [13]. Much of our current understanding of these systems uses molecular data that derive from experimental systems using transgene reporter assays [14] and harsh, chemical stress conditions [13]. This has led to some confusion over the frequency and transgenerational penetrance of stress-induced epigenetic phenomena. For instance, one report shows that UV-induced epigenetic control of somatic recombination can be stably inherited through at least four generations [9], whilst another reported no transgenerational effect of the same stress using the same reporter lines [13]. There is, nevertheless, clear evidence that wholesale epigenetic responses to stress can be carried between generations. For example, Boyko *et al*. [14] showed that exposure of parental *Arabidopsis* lines to a range of stresses (salt, UVC, cold, heat and flood) induced global increases in DNA methylation and raised tolerance to the same stresses in the immediate filial generation, but that the effects did not persist into subsequent generations. Exploitation of genetic experimental plant systems such as epigenetic recombinant inbred lines (epiRILs) [15] has also yielded considerable advances. For example, successive methyltransferase-deficient generations [9], differentially stress-sensitive RILs [3] and apomictic in-bred perennials [10] have all shown a convincing transgenerational effect, although the derived molecular data on patterns of epigenetic inheritance are complex and clear functional relationships have yet to be deduced. Nevertheless, it has been possible to demonstrate that enhanced resilience in these progeny is correlated with the ability to maintain symmetric (CG sequence context) DNA methylation [9,16] and requires the production of siRNAs at RNA-directed DNA methylation (RdDM) target loci [12,17]. Thus, whilst there is growing evidence of an association between inheritance of stress-induced epigenetic change and transgenerational conditioning of offspring to improve the response to the same stress, a clear functional link between the two remains elusive.

One appealing option is to focus attention on genes that are subject to epigenetic regulation and also play a functionally well-characterised role in development or environmental response. There are few, known marker genes of this sort. To date much experimental work has made use of the *Arabidopsis thaliana* flowering delay gene *FWA*[18] and more recent experiments with epigenetic regulation of the stress-sensitive UBP1b protein have also been reported [19]. Several epigenetically-regulated transposable and repetitive elements have also been used as markers, and for which the release of transcriptional gene silencing (TGS) has been observed in response to prolonged heat stress, UV-B and freezing [20–22]. Transgenerational transmission of this release was, however, transient, limited to differentiated tissues and was not correlated with changes in DNA methylation. The continuing need is for a gene whose expression is environmentally responsive, moderated by a heritable form of epigenetic control (DNA methylation), and has a direct and measurable impact on phenotype. We previously showed that stable DNA hypermethylation at two loci in the *A. thaliana* stomatal development pathway, *SPEECHLESS* (*SPCH*) and *FAMA*, was induced by growth in low relative humidity [23]. This additional methylation was correlated with a reduction in the expression of both genes, reduced stomatal index (SI; stomata as a percentage of epidermal cells) and an increase in the production of siRNAs [23]. Although RdDM was responsible for stress-induced methylation at both loci, there were differences between the two: At *SPCH* low humidity-induced methylation was associated with the induction of ~24 nt siRNAs at downstream helitron transposable elements (TEs), whereas at *FAMA* methylation spread from a 21 nt, genic, post-transcriptional gene silencing (PTGS) siRNA target [23]. In contrast with heat-inducible alterations in endogenous loci, which generally lead to hypomethylation of retroelements, these two genes are directly suppressed by additional DNA methylation and thus provide us with methylated targets in parents from which we are able to assess transmission to the progeny. In this study, we tested whether low humidity-induced methylation was transgenerational at these gene loci, and examined its persistence under repeated stress and association with local siRNAs. Remarkably, although methylation could be transmitted to the progeny and was associated with the stressed stomatal phenotype, methylation status and expression responses to repeated treatment were reversed rather than stabilized in the progeny.

## 2. Results

### 2.1. Inheritance of Stress-Induced DNA Methylation

We chose two target sequence fragments each of *FAMA* and *SPCH* to assess the parent*treatment effect on DNA methylation status and inheritance across generations. *FAMAa* is a genic site associated with a 21 nt siRNA target and the start point of low relative humidity (LRH)-induced methylation in the parent; *FAMAb* spans the transcriptional start site and a short section of approximately 90 bp immediately upstream of the gene sequence. Both fragments were highly methylated in all sequence contexts in LRH-treated parents (Table 1). *SPCHa* spans the transcription start site and contains a CT tandem repeat of 49 bp. This fragment was hypomethylated in all LRH-treated parents (Table 1). In contrast, *SPCHb* resides just downstream of the *SPCHa* target site but was hypermethylated in all contexts in LRH-treated parents (Table 1). In the same bisulfite-modified samples, the endogenous negative control (*SCREAM2)* was hypomethylated in all LRH parents (Table 1).

The methylated cytosines observed in the LRH parental plants were largely retained among the progeny grown under control conditions (LRH-control), with both fragments of *FAMA* and *SPCHb* remaining hypermethylated. Equally, the hypomethylated status of *SPCHa* or *SCREAM2* in the parents was unchanged in the offspring (Table 1). In *SPCHb,* methylation between generations was highly predictable (the inverse of entropy), consistent with direct inheritance of each of the methylation marks between generations (Table 1). The pattern was notably less clear in *FAMA* (Table 1). Although the entropy of *FAMAa* (0.08) was much less than of *FAMAb* (0.17), this lack of predictability of distribution meant that there was no statistical support for the hypothesis that methylation was directly inherited in either fragment of *FAMA*.

**Figure 1:**
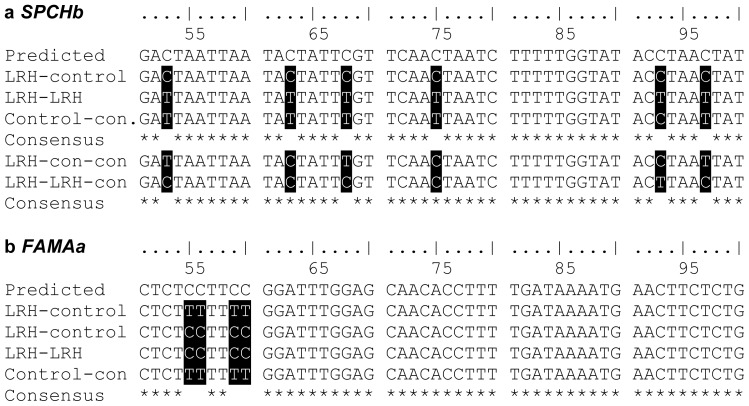
Example 50 bp fragments of (**a**) *SPCHb* and (**b**) *FAMAa* following bisulfite-sequencing of pooled sample DNA from progeny grown in the control and low relative humidity (LRH) treatment, parent-progeny. “Predicted” fragments are majority examples from the LRH-induced methylated parents. Differential methylation with progeny treatment is highlighted. When unmethylated, a bisulfite-sequenced “C” will convert to “T”. Two examples are shown for *FAMAa* control-grown progeny, where both methylated and unmethylated cytosines were found, to illustrate the contrast.

The pattern of methylation inheritance was markedly different when the progeny of parents grown in LRH were also exposed to the identical low humidity (LRH-LRH). Among these progenies, *FAMA* was strongly re-methylated at predictable sites identified in the parents, such that the effect of the treatment was highly significant (Table 1 and Figure 1), suggestive of consistent *de novo* methylation in response to LRH at this locus. However, parental LRH-induced hypermethylation at the *SPCHb* sites was lost from progeny exposed to identical LRH treatment (Table 1 and Figure 1). This surprising effect was reproducible and significant across three repeated experiments (χ^2^ < 0.001 for all replicates).

**Figure 2:**
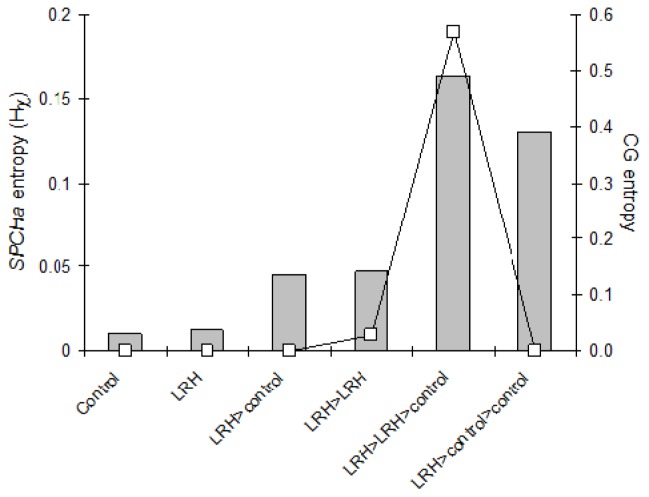
Mean entropy (H) of methylation at *SPCHb* with parental treatment*progeny treatment (parent > progeny > progeny) for three generations. The secondary *y* axis (open squares) shows mean entropy in symmetric (CG) sequence contexts.

In second generation progeny of methylated grandparents, methylated cytosines were found in all sequence contexts at *SPCHb.* However, the predictability of methylation was no longer significant in these plants (Figure 2) whether the parent was grown in control or LRH conditions (LRH-control-control and LRH-LRH-control), and the entropy of CG methylation always increased in LRH offspring exposed to LRH at *SPCHb* (Figure 2).

### 2.2. Gene Expression and the Abundance of siRNAs

We next examined the impact of treatments in previous generations on the abundance of siRNAs targeting *SPCH* and on *SPCH* expression. In comparison with second generation control plants (Control-control), expression of *SPCH* was reduced in the transgenerationally methylated progeny of LRH-treated parents (LRH-control) but increased in LRH-LRH plants, which had lost methylation (Table 2). The abundance of local siRNAs associated with the gene and helitron transposable elements >1 kb downstream of the gene model was positively correlated with methylation and negatively correlated with *SPCH* expression (Table 2). This correlation was still significant, but less reliable, for a unique, 21 nt, genic *SPCH* siRNA (*r*^2^ = 0.56, *p* = 0.03) when analyzed separately. In contrast, there was no relationship between *FAMA* expression and local 23–24 nt siRNAs (Table 2) but *FAMA* expression was reduced and increased (in LRH-control and LRH-LRH samples respectively) inversely correlated with abundance of a unique, 21 nt, genic siRNA (*r*^2^ = 0.85, *p* = 0.05).

### 2.3. sRNAs Are Lost When the Progeny of Stressed Parents Are Exposed to Repeated Stress

**Figure 3:**
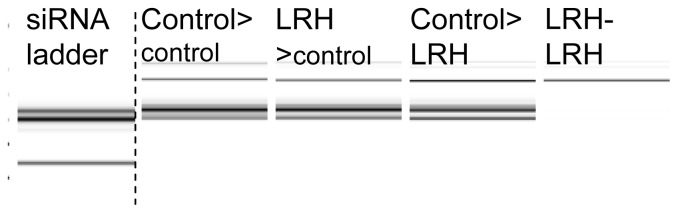
Example small RNA gel showing the concentration (μL^−1^) of 24 nt and 21 nt ds-RNAs in total RNA (200 ng μL^−1^) in the progeny of control and low relative humidity(LRH)-stressed seedlings (parent > progeny growth conditions).

We analyzed the abundance of all small RNAs (sRNAs) within each size class using total RNA extracts from progeny samples. There was an increase in sRNAs of all size classes on first exposure to the treatment, *i.e.*, control-LRH in comparison with control-control plants (*p* = 0.016, Figure 3). This general increase was less pronounced in transgenerationally methylated progeny (LRH-control, *p* = 0.098, Figure 3). Here, the significant increase was confined to the 24 nt sRNA class (*p* < 0.001), with no significant elevation of the 21 nt class (*p* = 0.149). At this level of sensitivity (200 ng unenriched total RNA), sRNAs of all size classes were almost undetectable in LRH-LRH plants (Figure 3). In samples enriched for the small RNA fraction, we were able to quantify specific siRNAs (as above) in LRH-LRH samples but, as a fraction of all sRNAs (15–150 nt), these were always scarce and there was a significant interaction between parent and treatment (*p* = 0.003).

### 2.4. Stomatal Phenotype under LRH Stress Is Heritable

**Figure 4:**
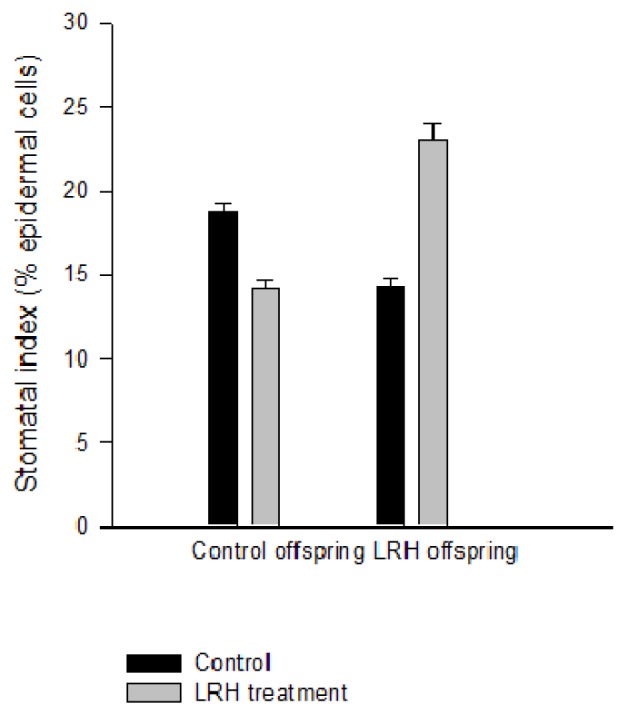
The mean (±SE) leaf stomatal index (SI; stomata as a percentage of epidermal cells mm^−2^) in the progeny of control and low relative humidity (LRH)-grown parents in the control (black bars) and LRH treatment (grey bars).

SI was invariably reduced among all progeny of reciprocal crosses, LRH ♀ × control ♂ or Control ♀ × LRH ♂, when compared with reciprocal Control × Control crosses (mean 24% decline, *p* < 0.001, *d* ≥ 0.80). SI was always reduced in the immediate progeny of LRH parents grown in control conditions (mean 26% decline, *d* ≥ 0.80) and increased in the same progeny in the LRH treatment (mean 40% increase, *d* ≥ 0.80) (Figure 4). The effect of the parent*treatment interaction was significant in each repeated experiment (Two-way ANOVA, Treatment *p* < 0.001, Parent *p* < 0.001, Interaction *p* < 0.04). SI of LRH-control-control plants was 17.5 (±0.9) and of LRH-LRH-control plants 11.94 (±0.9) indicating an effect of the treatment of the immediate progenitor on stomatal development (*p* = 0.03, *d* ≥ 0.80).

## 3. Discussion

The inheritance of epigenetic stress-tolerance is a matter of great interest and of continuing debate [4,24,25]. By assaying, in detail, the low humidity-induced stomatal phenotype, trangenerational patterns of methylation and siRNAs at stomatal developmental pathway gene loci we have been able to add some intriguing insights to augment current understanding of transgenerational inheritance in the model plant *Arabidopsis thaliana*.

Using isogenic seed stocks and reciprocally crossed plants varying only in treatment, we were able to induce additional DNA methylation at *SPCH* and *FAMA* with low relative humidity treatment; a finding consistent with our previous study [23]. This methylation was faithfully transmitted to progeny at *SPCH*(*b*) but less faithfully at *FAMA*(*a* and *b*). Methylation was never induced in a hypomethylated, repeated sequence fragment of *SPCH* (*SPCHa*) or at another gene in the same developmental pathway (*SCREAM2*) in the same samples. These observations indicate that the observed methylation was, indeed, transgenerational and targeted rather than an artefact of experimental procedures. Interestingly, despite the lack of any other known source of increased methylation, there was no statistical support for the transgenerational inheritance of low humidity-induced methylation at *FAMA* whereas there was at *SPCH*. Our results are consistent with those of Boyko *et al*. [14] who found stress-induced, transgenerational hyper- and hypo-methylation in one generation of *Arabidopsis* progeny, and suggest that the penetration and predictability of transgenerational stress-induced methylation vary between loci in *Arabidopsis*, as they also appear to do among apomictic dandelion plants [10]. They also imply that experimentation (as practiced here) may help shed light on previous conflicting results.

In repeated experiments and in all sample plants, the SI was reduced in LRH-control and increased in LRH-LRH in a manner that positively correlated with expression of *SPCH* and *FAMA* and inversely correlated with methylation of *SPCH*(*b*) and with the accumulation of siRNAs associated with both loci. Uncertainty regarding transgenerational methylation was greater in second generation progeny at *SPCH.* Here, in particular, there was a separation in CG, as opposed to any sequence context, methylation that was dependent on the environmental re-exposure of transgenerationally methylated progeny. The stability of the inherited *Arabidopsis* epigenome is dependent on CG methylation [16] and *Arabidopsis* therefore has mechanisms, involving 24 nt siRNAs, for re-establishing lost methylation that seemingly protects against transposable element reactivation [17]. Patterns created by recombined de- and re-methylation are locus-specific, relatively unstable and dynamic [26] but lead to a range of stress-tolerance and phenotypes similar to the natural range amongst *Arabidopsis* accessions within six to nine generations [26,27]. In our experiments, SI was restored to wild type levels by the second generation following methylation in the grandparent, and transgenerational reduction was once again correlated with the environmental experience of the immediate parent. One way to interpret these data is to adopt the reasoning of Boyko *et al*. [14] that the transgenerational stress-induced phenotype does not persist in successive generations and requires repeated exposure to the stress, consistent with previous results. Another interpretation, however, is that at some re-methylatable loci, the dynamic process of stress-induced de- and re-methylation can lead to opposite phenotypic effects in progeny; as, for example, was reported by Pecinka *et al*. [13] in progeny following salt and osmotic stress. Johannes *et al*. [15] similarly noted variability in correlation between methylation and trait status across generations and Tittel-Elmer *et al*. [22] noted that the release and restoration of TGS over time is dependent on the efficiency of the process. Our data imply that trait correlation with methylation also depends on the sampled generation and the growth conditions since any stress-induced *de novo* methylation. Our results indicate that it is likely that the process of demethylating and re-establishing methylation under low humidity following its inheritance at *SPCH* causes cohorts of nuclei that have different methylation status at mitotic reproduction [28]. An alternative explanation is that cytosines are protected from re-methylation following demethylation, as has been proposed previously [16], but that this protection is imperfect.

Although our results seem to add to the complexity of transgenerational, stress-induced, molecular epigenetic information, they do not imply a lack of general response or stochasticity as they were reproducible. The potential for epigenetic breeding for transgenerational resistance to stress in plants, therefore, clearly exists for the SI trait as for others [24,29] but its persistence may depend on a greater understanding of the epigenomic factors that affect the entire pathway and, in particular, the targeted stress responses of siRNAs. Characterization of the epigenetic flexibility of pathways such as the stomatal gene pathway should have resonance in predicting the impact of some abiotic stresses. From the perspective of crop production, greater understanding of the extent to which environmental transgenerational priming, as described here, applies elsewhere could have direct benefits in efforts to improve the interaction between agronomic practice, seed production and the enhancement of crop productivity. From a more practical standpoint, one potentially negative consequence of our findings is that care should be exercised in the design and interpretation of physiological experiments using seed stocks secured from parents grown in uncharacterized or unknown environments.

It is clearly difficult at this stage to predict the extent to which the findings described here will have a broader resonance on plant or biological science. On the one hand, there does seem to be reasonable conservation of the genes involved in the control of stomatal development but on the other, it seems questionable that there will be similar conservation of epigenetic regulation of *SPCH* seen here, given the importance of a close proximity to a helitron transposable element (TE) as a landing site for the siRNAs to drive methylation-based silencing in *Arabidopsis*[23]. Moreover, it is the pattern of transgenerational inheritance of methylation and its capacity to influence gene silencing based on parental exposure to stress that perhaps holds the greatest relevance for other biological systems. For instance, our study seems to suggest a predictive adaptive response (PAR) for stomatal development similar to that investigated in insect and mammalian organisms [30]. In this case the PAR is the setting of progeny stomatal phenotype to match the parental growing environment in anticipation that this same environment will be encountered in the next generation. The data we present for DNA methylation at *SPCH*(*b*) (Table 1) show that it is similar in both predictive environments (LRH-LRH and Control-control) and both unpredictive environments (LRH-control and control-LRH (parental)). Stomatal development has many parallels with cell fate specification in animals [31] and provides an attractive framework to investigate PARs. Specifically, the environmental effect on stomatal development (and what is known about stomata gene expression in response to environment; [32]) is systemic, *i.e.*, signalled from mature to young, developing leaves [33]. As far as is known, the response is immediate in *A. thaliana.* We have hypothesized [23] that the control of gene expression by siRNAs (RdDM) could form this systemic signal and explain phenotypic plasticity. A transgenerational continuation of this regulatory mechanism, as evidenced here, could indeed form a PAR and usually has a cost as well as an advantage in the predictive environment where increased water use efficiency (WUE) with fewer stomata leads to reduced growth rate and biomass and vice versa. These costs and benefits could be measured to separate plasticity from any PAR. It has been argued that the systemic signal for change in stomatal development with environment is itself changed WUE [34] (although our previous data on stomatal density [23] do not support this suggestion) which complicates studies. We believe, however, that the stomatal phenotypic responses reported here may have potential to help answer questions about PAR in plants in the future.

Our results show an inverse correlation of *SPCH* and *FAMA* expression with local siRNAs induced by low relative humidity treatment in the first generation of exposure to this stress. Our previous report detailed RdDM spread from downstream ~24 nt siRNAs at helitron TEs in *SPCH* and initiated at a genic 21 nt siRNA in *FAMA*[23]. Against a general background of increase in 24 nt siRNAs in LRH-control plants, and a marked reduction in all siRNAs when progeny were exposed to the same stress as their parents (LRH-LRH), abundance of these siRNAs was still correlated with methylation at these two loci. These results may provide some mechanistic insight for the differences in predictability of transgenerational methylation between the two loci. The epigenetic control of nearby TEs is generally considered deleterious for gene expression [35]. Bursts of transposition may, however, create new regulatory insertions responsive to environmental stress and may result from initial exposure to the stress [36]. The biogenesis of 24 nt siRNAs is required to prevent heat-stress induced transgenerational retrotransposition of the *ONSEN copia*-type retrotransposons and control is not restricted to the gametophytic phase [36]. In order to allow for the creation of new stress-responsiveness, at some point this epigenetic control must be relaxed [25]. We propose that, in the case of exposure to low relative humidity at least, a second exposure to the stress might provide such a relaxation point. In this way, it is possible that *SPCH* is a gene that maintains its transcriptional flexibility by co-opting the epigenetic selective control by siRNAs of its neighbouring transposable elements. It should be recognised, however, that the scope of the current study is strictly limited to methylation-associated regulation of *SPCH* and *FAMA* and so does not accommodate for the influence of other pathways that may impact on WUE (such as processes regulating stomatal opening) or of the contribution made by other epigenetic regulatory mechanisms.

## 4. Experimental Section

### 4.1. Plants and Growth Environment

**Figure 5:**
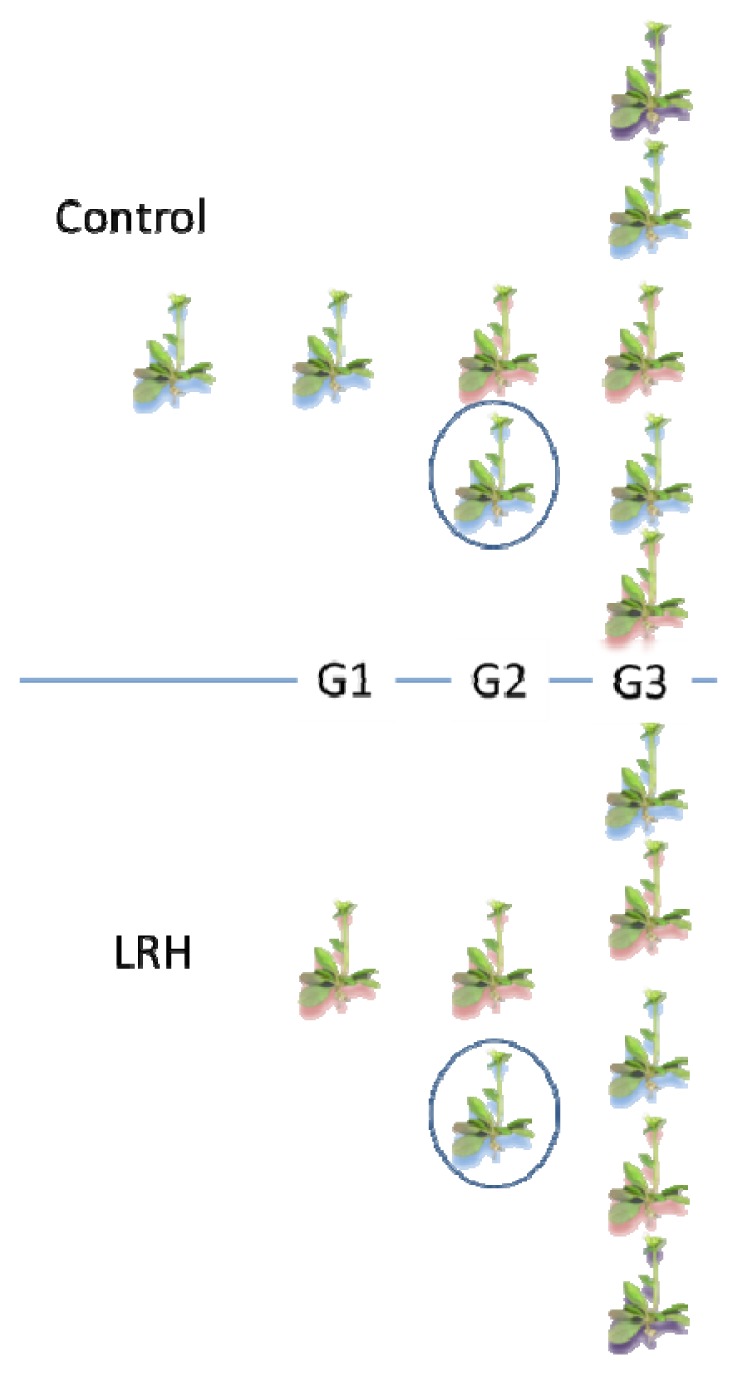
The experimental design. Supplied seeds from single seed descent were grown under controlled conditions (far left) and used as founder parents (G1) in the control growth cabinets (65% RH, top half of diagram) and low relative humidity (LRH, 45% RH, bottom half of the diagram). Selfed progeny of the control (blue background) and LRH (red background) plants were grown in each environment (G2) and selfed progeny of these G2 plants grown in each environment (G3). The progeny of plants reciprocally crossed between the control and LRH environments (purple background) during G2 (circled) were grown in each environment. The entire experiment was repeated three times by growing new supplied seeds in the control conditions at each generation, i.e. moving one to the right to start new lines.

*Arabidopsis thaliana* (L.) Heynh. Landsberg erecta (Ler) seeds from single-seed descent were supplied by NASC (Nottingham, UK). Sixty seeds each were germinated and grown in two controlled environment growth cabinets (Saxcil, R.K. Saxton, Bredbury, Cheshire, UK) until harvest with conditions set as in [23] and with the relative humidity of one cabinet controlled at 45% ± 5% (LRH) whilst the other was maintained at 65% ± 5% (control). At senescence (stage 9.70 [37]), seeds were harvested from each individual. Harvested seeds were measured using ImageJ software (Freeware, NIH) and homogenous harvested seeds from three control and three LRH-grown parents each were divided and grown alongside supplied seeds in control and LRH conditions (as described above). This experiment was repeated three times with self-pollinated plants to create three-generation lines (Figure 5). Additional growth chambers (Saxcil, as before, and Sanyo Gallenkamp, Loughborough, UK) were used in all repeated experiments to allow for growth chamber effects. In addition, reciprocal crosses were made between 12 plants (progeny of control parents) in each direction, in each environment, in two successive experiments.

### 4.2. DNA Methylation Analyses

DNA was extracted from whole seedlings (first true leaf stage) of ≥12 replicate progeny plants in each repeated experiment using the DNeasy plant mini-kit (Qiagen, Manchester, UK) according to the manufacturer’s instructions, and from whole seedlings and mature leaves of their individual parents described previously [23]. DNA cytosine methylation was assessed from 80 sub-cloned PCR amplicons of each fragment of each target gene following bisulfite-modification of genomic DNA exactly as described in [23], using the EZ DNA methylation kit (Zymo Research, Orange, CA, USA). Five nanomole per liter of labelled, synthetic DNA containing methylated and unmethylated cytosines was added to each DNA sample as a positive control spike for bisulfite conversion of each PCR product (Sigma-Aldrich Ltd., Gillingham, UK). Positive control and sample clone sequences were separated and aligned using a custom alignment program in Perl. Differential methylation was assessed with reference to the unmodified genomic DNA sequence and comparison of cytosine to thymine conversion between treatments.

### 4.3. RNA Analyses

Total RNA was isolated from progeny seedling samples, using the mirVana miRNA isolation kit (Ambion, Warrington, UK) according to the manufacturer’s instructions. All samples were checked and quantified using the Agilent Bioanalyzer RNA 6000 Nano chip and bioanalyzer (Agilent, Winnersh, UK) prior to use. sRNAs in each equalised total RNA sample (*n* ≥ 3) from each repeated experiment were quantified using the Small RNA chips and kits (Agilent, Winnersh, UK) and the “smear analysis” software function of the Agilent 2100 Bioanalyzer (as before) used to compare against small, dsRNA standards (New England Biolabs, Hitchin, UK) and the quantification standard ladder provided in the kit.

Multiple siRNAs and mRNA expression was analyzed by in solution hybridization and RNase digestion of both the total and the small RNA fractions that had been enriched from 200 ng of total RNA from each sample. This was achieved by hybridization using custom-synthesized probes, followed by electrophoretic separation and quantification of the protected (post-labelled fluorescent) probes using the Small RNA chip and bioanalyzer software as before.

### 4.4. Primer and Probe Designs

Primers for bisulfite-specific PCR were as described in [23] for target fragments of the *SCREAM2* (At1g12860), *FAMA* (At3g24140) and *SPCH* (At5g53210) genes. Antisense probes for *FAMA*, *SPCH* and local siRNAs were as in [23].

### 4.5. Stomatal Analyses

SI was determined by making impressions of the entire abaxial surface of one mature rosette leaf (insertion 6–8, length approximately 40 mm) from 48 plants (each of 16 replicate plants from each of the three individual parents in the descent lines) at the same physiological stage (6.50, [37]) in each repeated experiment. Digital images were then captured from an Axioskop 2 microscope with an Axiocam camera attached (Carl Zeiss Ltd., Cambridge, UK), using Axio Vision 3.1 (Image Associates, Oxfordshire, UK) software and the number of stomata and other epidermal cells per unit area counted using ImageJ software (as before).

### 4.6. Statistical Analyses

Statistical tests were conducted using Minitab v15 software (Minitab, Inc.: State College, PA, USA, 2009). To assess the predictability of transgenerational methylation, entropy plots for each column of each parent*treatment sample sequence alignment were created using BioEdit v. 7.0.9.0 [38] and mean entropy scores calculated for each position in a fragment. The number of methylated bases was calculated for each row of the alignment and the mean number calculated for each parent*treatment condition. Data were checked for homogeneity of variances using both Bartlett’s and Levene’s tests and normality using box-plots followed by Ryan-Joiner tests. One-sided χ^2^ goodness-of-fit tests were used to determine the statistical significance of the difference in distribution of methylated cytosines between the methylated parent and its progeny in the control conditions (inheritance) and between the control and LRH treatments (treatment) at α-levels of 0.05.

Because both parent and treatment could be considered explanatory, predictive variables in parent*treatment progeny plants, all other responses (dependent variables, *i.e.*, sRNA concentrations, mRNA and siRNA concentrations, stomatal indices) were tested in complete, factorial models with parent and treatment as fixed factors (by two-way ANOVA) following averaging of the technical replicate (seeds from one parent) responses and tests of normality and homogeneity as before. The effect size of the parent and treatment combinations on stomatal index for each repeated experiment (see experimental design Figure 5) was calculated and compared as Cohen’s *d*[39] using the pooled standard deviation of the means.

Differences in stomatal indices from reciprocally crossed plants varying only in treatment were analysed using Student’s *t*-tests in each of two repeated experiments and effect sizes calculated as before.

Measured expression levels of *SPCH* and *FAMA* and siRNA concentrations were inferred from the protected probes and modelled by drawing scatterplots of each (mRNA) response dependent on (siRNAs). Correlations were analysed by linear regression with adjusted *r*^2^ value and *P* value of ANOVA reported. *Post-hoc* Tukey’s pairwise comparisons were used to assess the significance of differences between target m/siRNAs, parents and treatments.

## 5. Conclusions

This study made use of parental stress-induced DNA methylation at two, known gene loci in a physiologically important pathway to investigate transgenerational epigenetic control of gene expression in response to abiotic stress. We found that both methylation and the stressed phenotype were heritable in reciprocal crosses from either genetic donor parent. There were differences in the direct inheritance of methylated sites between the two loci. These differences imply that the priming of individual genes, while targeted, is not the direct, mechanistic cause of transgenerational enhancement of resilience to abiotic stress. In contrast, the correlation between siRNAs, TEs and proximal gene methylation and expression in each generation indicates the importance of the siRNA biogenesis and RdDM pathways for potential pre-conditioning of progeny stress responses, and is consistent with previous results for their involvement in priming for improved defense against biotic stresses [40,41]. Therefore, our discovery that all siRNA classes are massively under-represented when progeny are challenged by the same abiotic stress experienced by their parents has implications for epigenetic breeding and might help explain the continuing transcriptional flexibility of plants’ responses to stress. It is, as yet, unclear whether this is a general response to all forms of stress and this will certainly be an important question to follow up. In the case of moderate water limitation under increased evaporative demand, it seems possible that by this molecular mechanism plants may “choose” to vary between generations the growth penalties associated with a more water use efficient phenotype and the faster growth associated with water use profligacy.

## Supplementary Information



## Figures and Tables

**Table 1:** The mean number of methylated cytosines (*C^m^) and entropy in selected 100 bp fragments of the SCREAM2*, *FAMA* and *SPCH* genes following bisulfite-sequencing of ≥80 amplicons for each fragment in pooled DNA from the parents exposed to low relative humidity (LRH) stress and in their progeny grown in control conditions (LRH-control) and in LRH (LRH-LRH) (*n* = 6 for each parent-progeny combination). The probability of the distribution of *C^m^being due to inheritance and/or the treatment is shown below; <0.001 = p* value, ns = not significant.

Methylated bases in 100 bp	*SCREAM2*	*FAMAa*	*FAMAb*	*SPCHa*	*SPCHb*
LRH (Parent)	0	17	14	0	30.2
LRH-control	0.1	13.5	12.9	0	29.2
*Entropy (H*χ*)*	0.004	0.08	0.17	0.001	0.02
LRH-LRH	0.13	16.6	13.5	0.03	11.4
*Entropy (H*χ*)*	0.003	0.05	0.05	0.001	0.02
Control-control	0	11.5	7.2	0	11.3
*Entropy (H*χ*)*	0.000	0.08	0.07	0.015	0.000
χ^2^ treatment	ns	<0.001	<0.001	ns	<0.001
χ^2^ inheritance	ns	ns	ns	ns	<0.001

**Table 2:** Mean concentration (pg μL^−1^) of *SPCH* and *FAMA* mRNA and their corresponding local siRNAs in whole seedling samples (6 in each of 3 repeated experiments) in the control and low relative humidity (LRH) treatment, parent-progeny (*n* = 9). Results of the linear regression for means of each biological replicate between genes and corresponding siRNAs are shown below with *post-hoc* comparisons; ns = not significant.

	*FAMA* mRNA	21–24 nt local siRNAs	*SPCH* mRNA	21–24 nt local siRNAs
LRH-control (pg μL^−1^)	244 (±52.6)	378 (±153.9)	511 (±74.5)	983 (±108)
LRH-LRH (pg μL^−1^)	541 (±128)	203 (±189)	1732 (±76.4)	312 (±74.7)
Control-control (pg μL^−1^)	334 (±54.3)	394 (±38.1)	809 (±134)	665 (±151)

**Linear regression**	***FAMA*and local siRNAs**		***SPCH*and local siRNAs**	
*r*^2^	0.34		0.87	
*p* value	0.875		0.013	

**Tukey’s pairwise comparisons**	**SE difference in means**		***p*value**	
Target RNA	104.7		ns	
Parent	110.3		0.017	
Treatment	106.1		ns	
