# Supplementary material for: Transgenerational, Dynamic Methylation of Stomata Genes in Response to Low Relative Humidity
Source: Int J Mol Sci. 2013 Mar 26;14(4):6674–89. doi: 10.3390/ijms14046674 (PMC3645660; doi:10.3390/ijms14046674)
Supplement: Supplementary file 1 [file ijms-14-06674-s001.docx]

Supplementary Information

**Figure S1.** Differential cytosine methylation with LRH stress at the *SPCH* locus
(TAIR v. 9.0). Hatched (blue) areas indicate methylated regions by methyl
capture + qPCR [23] in Chromosome 5 from 21584.3k to 21589.3k and bars methylated base pairs by 454 sequencing following bisulfite conversion (green = CG, blue = CHG, pink = CHH contexts). G1 plants (first exposure) plants are *de novo* methylated in all contexts under LRH stress. Progeny of these plants (G2) inherit the majority of methylation (LRH-control) but are substantially demethylated when returned to LRH stress (LRH-LRH). There is a loss of inherited methylation in the next generation (G3,
LRH-control-control) including at the upstream regulatory region and transcription start site, but this region is remethylated heritably during a second exposure to stress
(LRH-LRH-control). The (black) boxed region was assayed at individual base resolution by sub-cloning and sequencing samples following bisulfite conversion, and representative sequences are shown in Figure 1a for G2 and G3 and in [23] for G1.

© 2013 by the authors; licensee MDPI, Basel, Switzerland. This article is an open access article distributed under the terms and conditions of the Creative Commons Attribution license (http://creativecommons.org/licenses/by/3.0/).
